# A Novel Peptide Reagent for Investigating Disulfide-Coupled Folding Intermediates of Mid-Size Proteins

**DOI:** 10.3390/molecules28083494

**Published:** 2023-04-15

**Authors:** Nana Sakata, Yuri Murakami, Mitsuhiro Miyazawa, Shigeru Shimamoto, Yuji Hidaka

**Affiliations:** 1 Faculty of Science and Engineering, Kindai University, 3-4-1 Kowakae, Higashi-Osaka 577-8502, Japan; 2 Institute of Agrobiological Sciences, National Agriculture and Food Research Organization, Tsukuba 305-8634, Japan

**Keywords:** BPTI, cocoonase, disulfide, enzyme, folding intermediate, precursor, trypsin, zymogen

## Abstract

Investigations of protein folding have largely involved the use of disulfide-containing proteins, since the disulfide-coupled folding of proteins allows folding intermediates to be trapped and their conformations determined. However, studies of the folding mechanisms of mid-size proteins face several problems, one of which is that detecting folding intermediates is difficult. Therefore, to solve this issue, a novel peptide reagent, maleimidohexanoyl-Arg_5_-Tyr-NH_2_, was designed and applied to the detection of folding intermediates of model proteins. BPTI was chosen as a model small protein to estimate the ability of the novel reagent to detect folding intermediates. In addition, a precursor protein (prococoonase) of *Bombyx mori* cocoonase was used as a model mid-size protein. Cocoonase is classified as a serine protease and has a high homology with trypsin. We recently found that the propeptide sequence of prococoonase (proCCN) is important for the folding of cocoonase. However, it was difficult to study the folding pathway of proCCN since the folding intermediates could not be separated on a reversed-phase HPLC (RP-HPLC). Therefore, to separate the folding intermediates by RP-HPLC, the novel labeling reagent was used to accomplish this for proCCN. The results indicated that the peptide reagent allowed the intermediates to be captured, separated on SDS-PAGE, and analyzed by RP-HPLC without the occurrence of undesirable disulfide-exchange reactions during the labeling reactions. The peptide reagent reported herein is a practical tool for investigating the mechanisms of disulfide-coupled folding of mid-size proteins.

## 1. Introduction

Protein folding investigations have largely involved studies using disulfide-containing proteins, since the folding intermediates that are produced can be trapped and their conformations determined [1]. Folding mechanisms have been successfully determined for several proteins such as a bovine pancreatic trypsin inhibitor (BPTI) by investigating folding intermediates with mis-bridged or native disulfide bonds [2,3].

Cocoonase (CCN), a protein produced by the silkworm (*Bombyx mori*), consists of 226 amino acid residues (23.8 kDa) and possesses three intramolecular disulfide bonds [4]. Cocoonase is thought to specifically digest the sericin protein, which is the fibroin coating of silkworm cocoons, and has a high homology with trypsin [4,5,6,7]. In addition, cocoonase is biosynthesized as an inactive zymogen precursor protein as well as that of trypsin and is auto-catalytically or enzymatically processed into the bioactive mature form, cocoonase, by releasing an N-terminal propeptide (12 amino acid residues) [4]. We recently found that the CCN protein is kinetically trapped in a molten globule-like state at the final stage of the folding pathway and is converted into its native conformation with the assistance of the propeptide region [8]. To further investigate the role of the propeptide region in the disulfide-coupled folding of a precursor protein, prococoonase (proCCN), we carried out refolding and auto-processing reactions [9]. However, the refolding mixtures of proCCN contained several types of disulfide isomers that could not be completely separated by reversed-phase HPLC (RP-HPLC). Therefore, to obtain and separate the folding intermediates for studies of the folding mechanisms of mid-size proteins, a novel labeling reagent was developed and used to separate the refolding intermediates of the proCCN protein by RP-HPLC or SDS-PAGE.

In general, to investigate the folding mechanisms of disulfide-containing proteins, the folding intermediates are modified, separated, and identified by chemical reagents, RP-HPLC, and mass spectrometric analyses, respectively [2,3,10]. However, these methods are generally limited to small-size proteins (~10 kDa), such as BPTI (6.5 kDa), since the separation of folding intermediates of larger-size proteins on RP-HPLC or SDS-PAGE is frequently not possible. The precursor protein, prococoonase (25.1 kDa), possesses three intramolecular disulfide bonds and is classified as a mid-size protein; separating the folding intermediates of proCCN, which contain several types of disulfide pairings, by RP-HPLC is very difficult [11]. Therefore, we developed a novel peptide reagent for investigating intermediates that are produced during the disulfide-coupled folding of mid-size-size proteins, such as proCCN, as shown in Supplementary Materials Figure S1.

Several types of chemical reagents, including ICH_2_COOH and *N*-ethylmaleimide [12], which specifically react with a thiol group, are used to capture the folding intermediates of the disulfide-coupled folding of proteins [13,14]. Labeling reaction mixtures using ICH_2_COOH is often performed under highly alkaline conditions to accelerate the reaction, causing unfavorable thiol-disulfide exchange reactions. In this study, a maleimide group was employed, since the maleimide group reacts with a thiol group under mild conditions at near neutral pH values [12]. In addition, concerning the retention of folding intermediates by RP-HPLC, a hydrophilic poly-Arg peptide was designed to conjugate with hydrophobic folding intermediates. Moreover, arginine is thought to thermodynamically stabilize the exposed hydrophobic surfaces of folding intermediates during refolding reactions and effectively suppresses the formation of non-specific aggregates [15,16]. A Tyr residue was also added to the poly-Arg peptide to allow it to be retained on an RP-HPLC column and to increase the absorbance of the unit on RP-HPLC analyses. Maleimidohexanoyl-Arg_5_-Tyr-NH_2_ (Male-Arg_5_-Tyr-NH_2_) was designed to increase the molecular weight by 1 kDa after reacting with each thiol group for the separation of folding intermediates by SDS-PAGE analyses.

Thus, to study the folding mechanisms of mid-size proteins, the Male-Arg_5_-Tyr-NH_2_ reagent, which can react with the thiol groups of folding intermediates, was designed, chemically synthesized, and purified by RP-HPLC. To examine the utility of this reagent in analyses of folding intermediates, the peptide reagent was used to detect the folding intermediates of BPTI and proCCN as models of small- and mid-size proteins, respectively.

## 2. Results

### 2.1. Preparation of the Peptide Labeling Reagent, Maleimidohexanoyl-Arg_5_-Tyr-NH_2_ (Male-Arg_5_-Tyr-NH_2_)

In this study, to determine the folding pathways of mid-size proteins, a novel peptide reagent for labeling folding intermediates was prepared, as shown in Supplementary Materials Figure S1. The peptide labeling reagent was basically designed so as to increase hydrophilicity and molecular weight (1 kDa) and allow hydrophobic folding intermediates to be captured, as described in Introduction.

**Figure 1:**
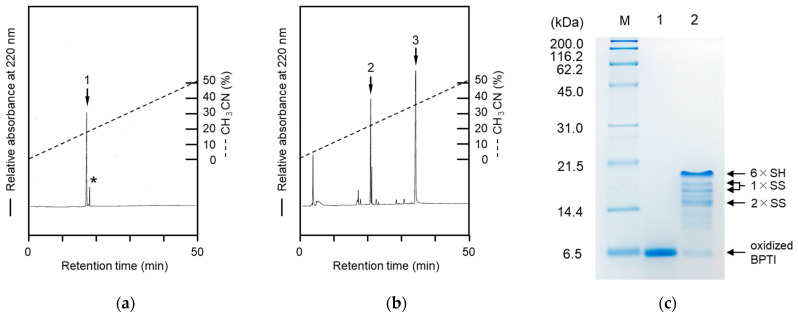
RP-HPLC profiles of the Arg_5_-Tyr-NH_2_ peptide (**a**) and reaction mixtures (**b**) of the Arg_5_-Tyr-NH_2_ peptide treated with *N*-succinimidyl 6-maleimidohexanoate. Tricine SDS-PAGE analyses (**c**) of the refolding solutions of BPTI. Peaks 1 and 2 were assigned to the Arg_5_-Tyr-NH_2_ and the Male-Arg_5_-Tyr-NH_2_ peptides, respectively, as evidenced by MALDI-TOF/MS. The asterisk and peak 3 represent an impurity and *N*-succinimidyl 6-maleimidohexanoate, respectively. Lanes 1 and 2 show the refolding solutions (1 h reaction) treated without or with the Male-Arg_5_-Tyr-NH_2_ reagent. “M” represents marker proteins (Nacalai Tesque, Kyoto, Japan). RP-HPLC analyses were performed using a Hydrosphere C_18_ column and the acetonitrile concentration was increased from 1% to 51% for 50 min.

The designed peptide was successfully synthesized by the Boc solid-phase method and purified by RP-HPLC after treatment with hydrogen fluoride, as shown in Figure 1a. The purified peptide was further treated with *N*-succinimidyl 6-maleimidohexanoate to introduce the functional group, a maleimide unit, for capturing the folding intermediates via the thiol group, as shown in Figure 1b. The target peptide was successfully obtained without side reactions. Mass spectrometric analyses of the purified Arg_5_-Tyr-NH_2_ and Male-Arg_5_-Tyr-NH_2_ peptides provided the observed monoisotopic mass values (*m*/*z*) of 960.9 Da and 1154.3 Da, respectively, which corresponded to the calculated mass values of 961.6 Da and 1154.9 Da, respectively.

### 2.2. Tricine SDS-PAGE Analyses of the Labeled Folding Intermediates of BPTI Using the Male-Arg_5_-Tyr-NH_2_ Reagent

To develop the labeling reagent for the folding analyses of proteins, folding experiments for BPTI (6.5 kDa) were performed as a model experiment, since the folding intermediates of BPTI have been extensively studied [3]. The refolding reactions of BPTI were performed under previously reported conditions [17]. The refolding mixtures were treated with the peptide reagent and then applied to Tricine SDS-PAGE to qualitatively detect the folding intermediates, as shown in Figure 1c. Protein ladder bands were clearly observed on the gel, confirming that the folding intermediates could be efficiently separated by Tricine SDS-PAGE. The bands for the proteins that had reacted with the peptide reagents were observed as proteins with higher molecular weights than expected because the positive charge of the poly-Arg residues affected the migration of the labeled proteins [18]. The observed protein bands were assigned to 6 × SH (fully reduced form with six peptide reagents), 1 × SS (with four peptide reagents), and 2 × SS (with two peptide reagents) proteins, based on the apparent molecular weights, as shown in Figure 1c. In addition, trace amounts of folding intermediates (containing unreacted thiol groups) with 1 × SS (containing one, two, or three peptide reagents) and 2 × SS (containing one peptide reagents) were also observed under the conditions used in this experiment. PEG-PCMal (Dojindo Laboratories, Kumamoto, Japan) was recently introduced to allow the redox states of proteins in cells to be visualized by SDS-PAGE analyses and the labeled proteins were well separated on the gels by the molecular weights related to the numbers of the thiol groups in the proteins [19,20]. The efficiency of the peptide labeling reagent, Male-Arg_5_-Tyr-NH_2_, was similar to that of the PEG-PCMal reagent, although the PEG-PCMal reagent is difficult to utilize in investigations of the folding mechanisms of proteins.

### 2.3. Labeling Reaction of the Folding Intermediates of BPTI Using the Male-Arg_5_-Tyr-NH_2_ Reagent

**Figure 2:**
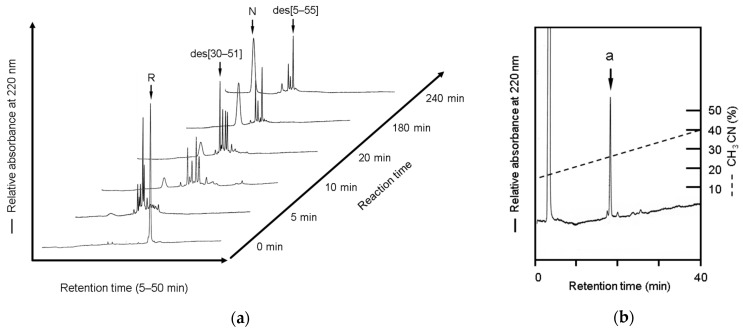
RP-HPLC analyses of the refolding mixtures at each reaction time point (**a**) and the reaction solution (**b**) of the des[5–55] intermediate treated with the Male-Arg_5_-Tyr-NH_2_ reagent. “R” and “N” represent the fully reduced and native forms of BPTI. Peak a in Figure 2b indicates the des[5–55] intermediate conjugated with the Male-Arg_5_-Tyr-NH_2_ reagent. RP-HPLC analyses were performed using a TSKgel Protein C_4_-300 column (Tosoh, Tokyo, Japan) and the acetonitrile concentration was increased from 5% to 20% for 15 min and subsequently from 20% to 70% for 25 min (**a**) and from 15% to 40% for 50 min (**b**).

To examine the issue of whether the peptide reagent was able to capture folding intermediates without disulfide shuffling, the refolding reactions of BPTI were performed and the folding intermediates were analyzed at each time point using RP-HPLC, as shown in Figure 2a. BPTI was used for the estimation of undesired disulfide shuffling reactions because the disulfide-coupled folding of BPTI has been extensively studied and the folding intermediates were easily separated by RP-HPLC [17]. The major folding intermediate, des[5–55], which contains two thiol groups and two disulfide bonds, was identified using the time-resolved folding reactions (Figure 2a) compared with that of the previous results [17]. The des[5–55] intermediate was then purified and confirmed by RP-HPLC (Supplementary Materials Figure S2) and mass spectrometry, respectively [3]. The purified des[5–55] intermediate was treated with the peptide reagent in 0.5 M Tris/HCl (pH 7.5) at rt for 1 h, as described in Materials and Methods. The des[5–55] intermediate yielded only a single product in the labeling reaction with a high recovery without any side reactions, as shown in Figure 2b. Importantly, no significant disulfide isomers were observed in the labeling reaction, indicating that the disulfide shuffling reaction can be excluded in the labeling reaction by the peptide reagent. Mass spectrometric analyses suggested that peak a (observed [M + H]^+^ = 8821.8 Da) in Figure 2b corresponded to the des[5–55] intermediate labeled with two peptide reagents (calculated [M + H]^+^ = 8826.4 Da), as summarized Table 1. Thus, the novel peptide reagent was able to sufficiently label the folding intermediate without disulfide bond shuffling (or scrambling) under the mild conditions used in this study.

### 2.4. SDS-PAGE Analyses of the Labeled Folding Intermediates of Prococoonase ([K8D]-proCCN′) Using the Male-Arg_5_-Tyr-NH_2_ Reagent

**Figure 3:**
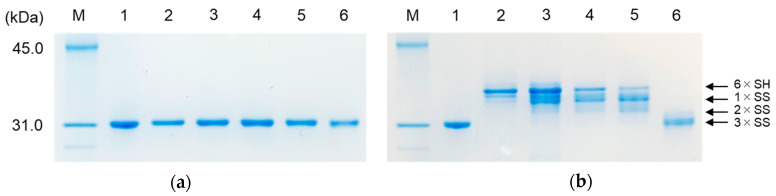
SDS-PAGE of the refolding mixtures of the [K8D]-proCCN′ protein. The refolding mixtures were treated with *N*-ethylmaleimide (**a**) or the Male-Arg_5_-Tyr-NH_2_ reagent (**b**) at each time point. The reaction mixtures (without treatments) were applied to lane 1. Lanes 2, 3, 4, 5, and 6 were the refolding solutions at the reaction times of 0, 1, 2, 3, and 24 h, respectively. The folding intermediates are indicated by arrows.

To apply the novel peptide reagent for investigating the folding mechanisms of mid-size proteins, we utilized the peptide reagent to capture the folding intermediates of prococoonase (25.1 kDa). For this purpose, a degradation-suppressed prococoonase, [K8D,K63G,K131G,K133A]-proCCN ([K8D]-proCCN′), was employed to avoid non-specific degradation during the refolding reactions [9]. To estimate the effect of the peptide moiety on separation on SDS-PAGE, *N*-ethylmaleimide was compared to the novel peptide reagent. The refolding reactions of the [K8D]-proCCN′ protein were conducted under the same conditions as that of BPTI and the folding intermediates were labeled with *N*-ethylmaleimide or the peptide reagent. The labeled reaction mixtures were analyzed by SDS-PAGE, as shown in Figure 3. As predicted, the several types of folding intermediates labeled with *N*-ethylmaleimide migrated together and were not separated by SDS-PAGE analyses, as shown in Figure 3a. In contrast, in the case of the peptide reagent, the folding intermediates, which contained 6 × SH (fully reduced form with six peptide reagents), 1 × SS (with four peptide reagents), 2 × SS (with two peptide reagents), and 3 × SS proteins (including disulfide isomers), were clearly separated well as ladder bands by SDS-PAGE analyses, as shown in Figure 3b. Thus, as expected, the peptide labeling reagent functioned efficiently and allowed several types of folding intermediates of the mid-size protein [K8D]-proCCN′ to be captured and separated. It should be noted that the time course analyses of the refolding reaction of the [K8D]-proCCN′ protein practically permitted the chase of the folding intermediates by SDS-PAGE analyses (Figure 3b), thus providing important information related to the folding mechanism of prococoonase.

### 2.5. RP-HPLC Analyses of the Labeled Folding Intermediates of Prococoonase ([K8D]-proCCN′) Using the Male-Arg_5_-Tyr-NH_2_ Reagent

**Figure 4:**
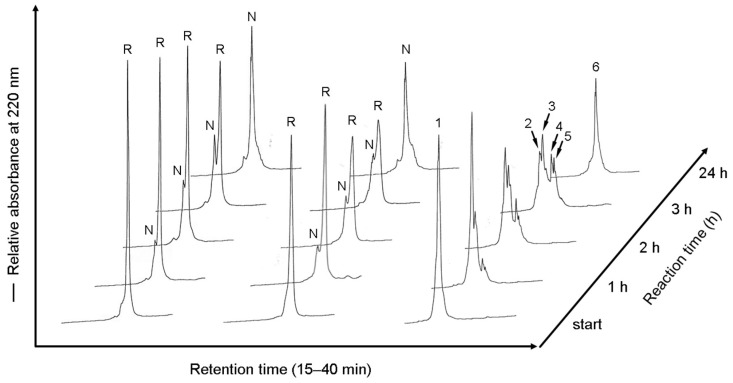
RP-HPLC profiles of refolding mixtures of the [K8D]-proCCN′ protein. Reaction mixtures without treatments are shown in the left lane. The refolding mixtures were treated with *N*-ethylmaleimide (middle) or the Male-Arg_5_-Tyr-NH_2_ reagent (right) at each time point. “R” and “N” represent the fully reduced and native forms of the [K8D]-proCCN′ protein. RP-HPLC analyses were performed using a TSKgel Protein C_4_-300 column and the acetonitrile concentration was increased from 27.5% to 40% for 25 min. Peaks 1–5 (right column) were determined by mass spectrometric analyses, as summarized in Table 1.

Finally, to examine the issue of whether the peptide reagent can be used for investigating the folding mechanisms of mid-size proteins by RP-HPLC analyses, the folding intermediates were labeled with the reagents at each reaction time to obtain further information regarding the folding pathway of proCCN, as shown in Figure 4. When *N*-ethylmaleimide was used for reacting with thiol groups, the folding intermediates, as well as that of the non-labeled folding intermediates, were not completely separated in RP-HPLC analyses, as shown in the middle and left lanes in Figure 4, respectively. As we expected, the separation of the folding intermediates of the [K8D]-proCCN′ protein on RP-HPLC analyses was greatly improved by labeling with the peptide reagent, compared with that for *N*-ethylmaleimide, as shown at the right lane in Figure 4. The mass spectrometric analyses of the numbered peaks in Figure 4 indicated that peaks 1, 2 and 3, 4 and 5, and 6 corresponded to the 6 × SH, 1 × SS, 2 × SS, and 3 × SS intermediates of the [K8D]-proCCN′ protein, respectively. The molecular masses of the proteins are summarized in Table 1. It should also be noted that the folding intermediates of the [K8D]-proCCN′ protein were sufficiently recovered during the refolding and labeling reactions by the peptide reagent, indicating that the peptide reagent allows hydrophobic intermediates to be retained as soluble forms. Importantly, the peptide reagent allowed several types of folding intermediates of the mid-size protein, [K8D]-proCCN′, to be separated in RP-HPLC analyses. The number of disulfide bonds in the folding intermediates of the [K8D]-proCCN′ protein increased depending on the reaction time, as shown at the right lane in Figure 4, although the disulfide pairings were not determined yet. The results of RP-HPLC analyses of the folding intermediates labeled with the peptide reagent at each reaction time were compatible with that of the SDS-PAGE analyses. Detailed analyses of the labeled folding intermediates will provide important information concerning the folding mechanism of proCCN, since RP-HPLC analyses generally provide more information related to the folding pathways of proteins than SDS-PAGE analyses. Thus, the use of the peptide reagent permitted the folding intermediates to be separated on SDS-PAGE and analyzed by RP-HPLC and will therefore be useful in practical investigations of the folding mechanisms of mid-size proteins.

## 3. Discussion

Investigations of protein folding pathways require a redox reagent for disulfide shuffling, a chemical reagent for labeling folding intermediates, and an instrument for their separation [14]. The redox reagents, such as glutathione, have been used to accelerate disulfide-coupled folding reactions of proteins to avoid the precipitation of hydrophobic folding intermediates. To address this issue, we previously introduced a redox reagent, 2-(diisopropylamino)ethanethiol (DPAET), that (based on the positive charge of the redox reagent) permits refolding reactions to be greatly accelerated [21,22]. Based on our hypothesis, several types of the positively charged redox reagents were synthesized and, when applied to refolding reactions, a high refolding recovery was obtained [23], although DPAET still shows the highest ability for accelerating refolding reactions among these types of reagents. However, labeling reagents that are designed to capture and separate folding intermediates of proteins have not been extensively studied, especially for mid-size proteins.

In this study, a peptide reagent for labeling folding intermediates of mid-size proteins was developed to study the folding mechanisms in which RP-HPLC and SDS-PAGE analyses are used as analytical methods. Several types of the labeling reagents have been used in studies of folding reactions of small-size proteins and these have significantly contributed our understanding of the nature of folding intermediates [14]. However, the small molecules, such as *N*-ethylmaleimide, are not applicable for use in mid-size proteins, since the separation of the folding intermediates cannot be completely separated on SDS-PAGE or determined by RP-HPLC, as shown in this study. PEG-PCMal was recently introduced for use in the analysis of the redox states of proteins in cells by capturing the thiol groups of the proteins on SDS-PAGE analyses. However, the PEG-PCMal reagent is designed only to consider the molecular weight for separating proteins on SDS-PAGE analyses, although the reagent may be applicable to investigations of protein folding. Therefore, to further develop a thiol capturing reagent for studying protein folding, the Male-Arg_5_-Tyr-NH_2_ reagent was designed to retain the solubility of the intermediates during the refolding reaction and to improve the separation of hydrophobic folding intermediates on RP-HPLC analyses. As we expected, the designed labeling peptide substantially improved the separation of the folding intermediates of the [K8D]-proCCN′ protein when a C_4_ column was used for the RP-HPLC analyses (Figure 4), thus permitting the folding mechanism to be revealed. Thus, the peptide reagent resulted in improved separation on RP-HPLC analyses that could be further improved by adding Arg residues to the peptide moiety.

Prococoonase was examined as a model protein for the analysis of folding intermediates derived from mid-size proteins. As described in Introduction, cocoonase is a member of the serine protease family and shares a high homology with trypsin [5,6,7,24]. Folding intermediates of zymogen proteins are generally difficult to separate by RP-HPLC, since degradation and aggregation occur during the refolding and activation reactions. The degradation that occurs during the refolding reaction of trypsin and trypsinogen complicates the folding analyses, even though investigating the folding mechanisms of serine proteases would be extremely interesting regarding the convergent enzyme evolution [25]. To address these issues, degradation-suppressed prococoonase, [K8D]-proCCN′ [9], was recently prepared and applied to examining the refolding reaction in this study. Importantly, significant levels of degradation products were not observed on RP-HPLC analyses, indicating that the refolding reactions of the protein occurred without degradation and that sufficient levels of intermediates had been labeled with the peptide reagent, as shown in Figure 4. Thus, the [K8D]-proCCN′ protein provided a breakthrough in terms of studying the folding mechanism of the trypsin superfamily [26]. Analyses of the folding of the [K8D]-proCCN′ protein will provide useful information related to not only the folding mechanism but also the folding of the convergent enzyme evolution of serine proteases, such as chymotrypsin and thrombin.

The refolding mixtures of the [K8D]-proCCN′ protein were labeled with the peptide reagent and analyzed by SDS-PAGE and RP-HPLC at each time point. Cocoonase possesses three intramolecular disulfide bonds that function to maintain the thermodynamically stable conformation. Theoretically, three disulfide bonds provide 45 possibilities (15 for 1 × SS, 15 for 2 × SS, and 15 for 3 × SS) for the combination of thiol groups and disulfide bonds in the folding intermediates. However, the number of actually observed folding intermediates was obviously less than the theoretical value. Disulfide bond formation of the folding intermediates of the [K8D]-proCCN′ protein was dependent on the reaction times, as shown in Figure 3b and Figure 4. Therefore, based on the obtained results, we speculate that the disulfide bond formation of prococoonase occurs in a stepwise manner via the formation of specific intermediates in the folding pathway.

In conclusion, the maleimidohexanoyl-Arg_5_-Tyr-NH_2_ reagent was prepared and utilized to capture the folding intermediates of a mid-size protein. The labeling reactions occurred without undesirable disulfide bond shuffling under mild conditions (near neutral pH). The labeled intermediates of the [K8D]-proCCN′ protein were separated well and detected by RP-HPLC and mass spectrometric analyses, respectively. Therefore, the novel peptide reagent for capturing and labeling folding intermediates will provide important information regarding the mechanism of folding of not only cocoonase but also of the trypsin super family.

## 4. Materials and Methods

### 4.1. Materials

Boc-amino acids, HBTU, HOBt, and glutathione were purchased from the Peptide Institute, Inc. (Osaka, Japan). BPTI, *N*-ethylmaleimide, and *N*-succinimidyl 6-maleimidohexanoate were purchased from GE healthcare (Chicago, IL, USA), Nacalai Tesque (Kyoto, Japan), and Tokyo Chemical Industry Co., Ltd. (Tokyo, Japan), respectively. All chemicals and solvents used were of reagent grade.

### 4.2. Preparation of Maleimidohexanoyl-Arg_5_-Tyr-NH_2_ (Male-Arg_5_-Tyr-NH_2_)

The protected peptide Boc-(Arg(Tos))_5_-Tyr(Cl_2_-Bzl)-NH_2_ was chemically synthesized on a Merrifield resin using HBTU/HOBt as the condensation reagent by the Boc solid-phase method [27,28]. After treatment with anhydrous hydrogen fluoride, the target peptide was purified by RP-HPLC (Figure 1a), as described below, and characterized by MALDI-TOF/MS and amino acid analyses [29,30]. The purified Arg_5_-Tyr-NH_2_ reagent (500 nmol) was dissolved in 0.1 M *N*-methylmorpholine (100 μL) and mixed with *N*-succinimidyl 6-maleimidohexanoate (1 μmol) at rt for 1 h. The reaction proceeded quantitatively, as shown in Figure 1b. The resulting product, Male-Arg_5_-Tyr-NH_2_, was separated and purified by RP-HPLC. The peptides were identified by mass spectrometric and amino acid analyses [28].

### 4.3. Reduction of the Proteins

A [K8D,K63G,K131G,K133A]-proCCN, denoted as [K8D]-proCCN′, was used in this study to suppress degradation during refolding reactions [9]. The oxidized form of the [K8D]-proCCN′ protein was prepared by a previously reported method [9]. The reduced forms of BPTI and the [K8D]-proCCN′ proteins were prepared by treatment with dithiothreitol as previously described, with minor modifications [8,17]. Briefly, the [K8D]-proCCN′ protein (5 nmol) was dissolved in 0.1 M Tris/HCl buffer (pH 8.0) containing 0.1 M dithiothreitol and 8 M urea and the solution was maintained at a temperature of 50 °C for 30 min. The reduced proteins were directly applied to RP-HPLC, purified, and stored at –80 °C until used.

### 4.4. Labeling Reactions of the Folding Intermediate Des[5–55] of BPTI by the Peptide Reagent (Male-Arg_5_-Tyr-NH_2_)

The refolding experiments of BPTI were performed under previously reported conditions [17]. The folding intermediate, des[5–55], was purified after 4 h of the reaction, lyophilized in vacuo, and dissolved in 8 M urea in a 0.05% solution of TFA (20 μL). To completely solubilize the intermediate proteins, 8 M urea was used. The protein solution (approximately 0.5 nmol) was mixed with the peptide reagent (5 nmol) in 0.5 M Tris/HCl buffer (30 μL, pH 7.5) containing 8 M urea, allowed to stand for 1 h at rt, and applied to RP-HPLC. The purified peptide was identified by mass spectrometry.

### 4.5. Direct Labeling Reactions of the Refolding Intermediates of Proteins with the Peptide Reagent (Male-Arg_5_-Tyr-NH_2_)

The refolding reactions of proteins were typically carried out in the absence of glutathione at rt. The protein (5 nmol) was dissolved in 8 M urea/0.05% TFA (50 μL) and diluted with 0.2 M Tris/HCl buffer (50 μL, pH 8.0) for the refolding reaction. We employed a relatively high concentration of urea (4 M) for the refolding reaction in order to allow folding intermediates to be completely captured. At each time point, aliquots (20 μL) of the reaction solution were treated with the labeling reagents (15 nmol), *N*-ethylmaleimide, or the Male-Arg_5_-Tyr-NH_2_ reagent, in 0.5 M Tris/HCl buffer (10 μL, pH 7.5) containing 4 M urea at rt for 10 min. The reaction mixtures were analyzed by RP-HPLC or SDS-PAGE. The electrophoresis of the refolding solutions of prococoonase and BPTI were carried out using SDS-PAGE and Tricine SDS-PAGE, respectively [31].

### 4.6. Reversed-Phase High Performance Liquid Chromatography (RP-HPLC)

A Waters M600 multisolvent delivery system (Bedford, MA, USA) and a HITACHI ELITE LaChrom system L2130 (Tokyo, Japan) equipped with a Hitachi L-3000 detector and a D-2500 chromato-integrator were used for the separation of the peptides and proteins, respectively. Peptides and proteins were separated by RP-HPLC using a Hydrosphere C_18_ column (4.6 × 150 mm, YMC Co., Kyoto, Japan) and a TSKgel Protein C_4_-300 column (4.6 × 150 mm, Tosoh Co., Tokyo, Japan), respectively. The peptides and proteins were eluted with a linear gradient of CH_3_CN in 0.05% TFA/H_2_O at flow rate of 1.0 mL/min at 40 °C. The eluates were monitored for absorbance at 220 nm and confirmed by MALDI-TOF/MS [29].

## Figures and Tables

**Table 1:** The mass values of the folding intermediates * of BPTI and [K8D]-proCCN′.

des[5–55]	**Peak ^†^**	**[M + H]^+^_calcd._**	**[M + H]^+^_obs._**	**Number of Thiol and** **Disulfide Bond(s)**	**Number of the Peptide Reagent(s)**
	a	8826.4 Da	8821.8 Da	2 × SH, 2 × SS	2
[K8D]-proCCN′	**Peak ^‡^**	**[M + H]^+^_calcd._**	**[M + H]^+^_obs._**	**Number of Thiol and** **Disulfide Bond(s)**	**Number of the Peptide Reagents**
	1	31,927 Da	31,933 Da	6 × SH	6
	2	29,616 Da	29,612 Da	4 × SH, 1 × SS	4
	3	29,616 Da	29,596 Da	4 × SH, 1 × SS	4
	4	27,304 Da	27,299 Da	2 × SH, 2 × SS	2
	5	27,304 Da	27,260 Da	2 × SH, 2 × SS	2
	6	24,992 Da	24,969 Da	3 × SS	0

* The folding intermediates treated with the Male-Arg_5_-Tyr-NH_2_ reagent. ^†^ Peak a was purified by RP-HPLC, as shown in Figure 2b. ^‡^ The numbers correspond to the peak numbers of the labeled folding intermediates of prococoonase on RP-HPLC as described below.

## Data Availability

Not applicable.

## Supplementary Materials

The following supporting information can be downloaded at: https://www.mdpi.com/article/10.3390/molecules28083494/s1, Figure S1: Strategy for investigating the folding mechanisms of mid-size proteins. The refolding mixtures are labeled with the Male-Arg_5_-Tyr-NH_2_ reagent and separated by SDS-PAGE or RP-HPLC. The number of disulfide bonds of the folding intermediates purified by RP-HPLC are determined by mass spectrometric analyses; Figure S2: The purified des[5–55] intermediate was rechromatographied under the same conditions as described in Figure 2b.

## Abbreviations

Boc, *tert*-butyloxycarbonyl; BPTI, bovine pancreatic trypsin inhibitor; CCN, cocoonase; CCN′, [K63G,K131G,K133A]-CCN; DPAET, 2-(diisopropylamino)ethanethiol; GSH and GSSG, reduced and oxidized forms of glutathione, respectively; HBTU, hexafluorophosphate benzotriazole tetramethyl uranium; HOBt, 1-hydroxybenzotriazole; MALDI-TOF/MS, matrix-assisted laser desorption/ionization time of flight mass spectrometry; RP-HPLC, reversed-phase high-performance liquid chromatography; rt, room temperature; TFA, trifluoroacetic acid.
