# Supplementary material for: A Novel Peptide Reagent for Investigating Disulfide-Coupled Folding Intermediates of Mid-Size Proteins
Source: Molecules. 2023 Apr 15;28(8):3494. doi: 10.3390/molecules28083494 (PMC10142513; doi:10.3390/molecules28083494)
Supplement: Supplementary file 1 [file molecules-28-03494-s001.zip › molecules-2323561-supplementary.pdf]

Supplementary Materials

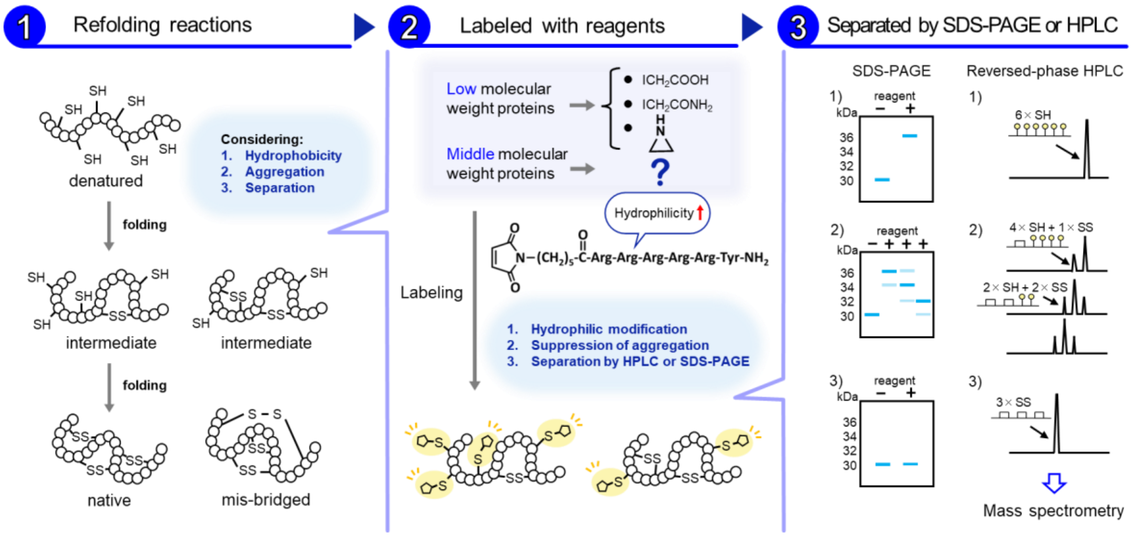

Figure S1. Strategy for investigating the folding mechanisms of mid-size proteins. The refolding mixtures are labeled with the Male-Arg5-Tyr-NH2 reagent and separated by SDS-PAGE or RP-HPLC. The number of disulfide bonds of the folding intermediates purified by RP-HPLC are determined by mass spectrometric analyses.

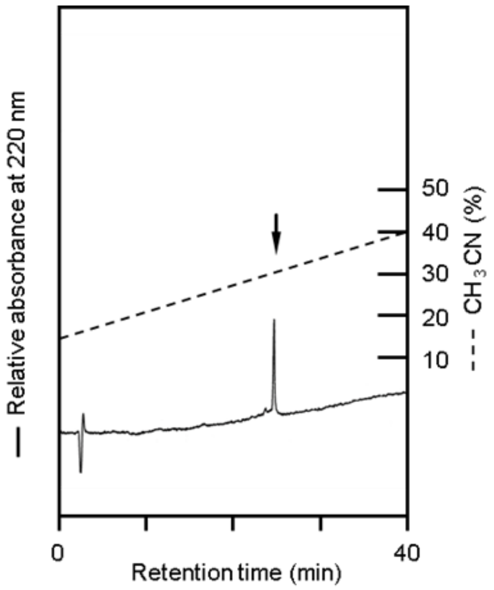

Figure S2. The purified des[5–55] intermediate was rechromatographied under the same condition as described in Figure 2b.
